# The role of polyreactive memory B cells in systemic lupus erythematosus

**DOI:** 10.1093/intimm/dxae058

**Published:** 2024-11-08

**Authors:** Keishi Fujio, Toshiyuki Ushijima, Tomohisa Okamura, Mineto Ota

**Affiliations:** Department of Allergy and Rheumatology, Graduate School of Medicine, The University of Tokyo, Tokyo 113-8655, Japan; Department of Allergy and Rheumatology, Graduate School of Medicine, The University of Tokyo, Tokyo 113-8655, Japan; Department of Allergy and Rheumatology, Graduate School of Medicine, The University of Tokyo, Tokyo 113-8655, Japan; Department of Functional Genomics and Immunological Diseases, Graduate School of Medicine, The University of Tokyo, Tokyo 113-8655, Japan; Department of Allergy and Rheumatology, Graduate School of Medicine, The University of Tokyo, Tokyo 113-8655, Japan

**Keywords:** autoantibody, polyreactive antibody

## Abstract

In systemic lupus erythematosus (SLE), the production of autoantibodies is a crucial characteristic, and B cells play a significant role in its pathogenesis. B cells are the immune cells most associated with the genetic predispositions of SLE, and recent clinical studies showing that anti-CD19 chimeric antigen receptor (CAR)-T cell therapy induces drug-free remission have underscored the importance of B cells in SLE. Meanwhile, various B-cell subsets exist across different stages of differentiation, from naive B cells to plasma cells, and identifying the important subpopulations within SLE remains a critical future challenge. Years of B-cell repertoire analyses have revealed the importance of polyreactive B-cell receptors (BCRs) and autoantibodies that react to various self-antigens and microbial antigens. Particularly, memory B cells with polyreactive BCRs, which play a crucial role in biological defense during the fetal stage, are characteristically differentiated in SLE. Type I interferon-mediated expression of CXCL13 and IL-21 in CD4^+^ T cells is associated with the development of polyreactive memory B cells. The expansion of the polyreactive B-cell repertoire, vital for defending against infections such as viruses, may exert an intrinsic function in SLE.

## Teaser

Antibody polyreactivity and its role in SLE

## Introduction

An important characteristic of immune abnormalities in systemic lupus erythematosus (SLE) is the production of autoantibodies. Specific autoantibodies, such as anti-double-stranded DNA (anti-dsDNA) antibodies, anti-ribonucleoprotein (RNP) antibodies, and anti-Smith (Sm) antibodies, are associated with disease activity and organ damage in SLE. Immunologically, SLE is characterized by the breakdown of immune tolerance to nuclear antigens (1, 2). Previous studies on SLE, including genome-wide association studies (GWAS) and gene expression studies in peripheral blood mononuclear cells (PBMCs), revealed a pivotal role of type I interferon (IFN) signaling in SLE (3). Elevated levels of type I IFN in the blood of patients with lupus (4) and the demonstration of a broad type I IFN-induced gene signature in SLE PBMCs provided compelling evidence that this cytokine family plays a dominant role (5). Moreover, type I IFN induces the development of plasmablasts (PB) when combined with Toll-like receptor (TLR) stimulation (6). However, why immune responses are established against these particular antigens among all nuclear antigens has remained a fundamental question for many years. Additionally, anti-phospholipid antibodies frequently appear in SLE, but why immune responses to phospholipids found in the membranes of live and dead cells are specific to SLE remains unclear. Accumulating evidence suggests a link between the reactivity of these autoantibodies in SLE and the feature of antibodies known as polyreactivity.

## Cross-reactivity of anti-dsDNA antibodies

Among the autoantibodies detected in SLE, anti-dsDNA antibodies are the most extensively studied in terms of their molecular characteristics. Studies have shown that anti-dsDNA antibodies exhibit cross-reactivity. Halpern *et al*. (7) first identified an idiotype marker of anti-DNA antibodies in patients with SLE. Furthermore, it has been demonstrated that anti-DNA antibodies share common features, including idiotype specificity, with antibodies against pneumococcal polysaccharides (8). Cross-reactive antibodies binding both bacterial antigens and dsDNA can be isolated from both humans and mice (9). The clonal relationship between anti-DNA antibodies and anti-microbial antibodies has been suggested based on the knowledge that anti-pneumococcal antibodies can be converted into pathogenic anti-DNA antibodies by a single nucleotide change (10). This finding elucidated the connection between the production of anti-dsDNA antibodies and immune responses to microorganisms. Additionally, DeGiorgio *et al*. (11) reported that epitopes of brain glutamate receptors bind to 50% of anti-DNA antibodies in patients and mice with SLE, and this binding may destroy neurons. This result suggests that cross-reactivity of autoantibodies might be related to specific organ damage.

B-cell receptors (BCRs) contain immunoglobulin heavy chains (IgH) and light chains (IgL) and are constructed through the recombination of V, D, and J genes. Human BCRs are formed by the random selection of approximately 40 types of V genes, 23 types of D genes, and 6 types of J genes. Generally, the complementarity-determining region 3 (CDR3) region, corresponding to the junctions of these genes, is considered crucial for determining antigen specificity, and the involvement of V genes in antigen specificity remains poorly understood. However, Richardson *et al*. found that about 10% of anti-dsDNA antibodies utilize the V-gene, *IGHV4-34*. Moreover, using the anti-idiotype antibody 9G4 against IGHV4-34, recombinant antibodies were produced from the BCRs of SLE memory B cells expressing IGHV4-34. These recombinant antibodies, which possess antinuclear antibody properties, could bind to DNA, dead cells, and cardiolipin (12).

These findings suggest that anti-dsDNA antibodies in SLE include those that show reactivity to both self-antigens, such as DNA and cardiolipin, and microorganisms, encompassing both autoreactivity and polyreactivity.

## Molecular characteristics of IGHV4-34

Human autoantibodies with IGHV4-34 bind to poly-*N*-acetyllactosamine carbohydrates (I/i antigens) on red blood cells and B cells, causing cold agglutinin disease and accounting for 5% of anergic naive B cells (13). Essentially all cold agglutinins from patients with this disease show reactivity with 9G4 (14). Reed *et al*. analysed the specificity of three IGHV4-34^+^ immunoglobulin G (IgG) antibodies isolated from healthy donors immunized with either the foreign *Macaca mulatta* D alloantigen or vaccinia virus. Each IgG was expressed and analysed in its hypermutated form after reverting each antibody to its unmutated germline sequence. In all cases, germline IGHV4-34^+^ IgGs are strongly bound to normal human B cells bearing I/i antigens. Self-reactivity was paradoxically removed by a single somatic mutation that decreased binding to foreign immunogens, whereas other mutations increased foreign-antigen binding. These data indicate that mechanisms of somatic mutation exist in humans to escape germline-encoded self-reactivity (15). The germline *IGHV4-34* gene segment encodes inherently self-reactive antibodies that recognize I/i carbohydrates expressed by red blood cells through a specific motif in framework region 1 (FWR1).

IGHV4-34^+^ clones are common in the naive B-cell repertoire but are rarely observed within the IgG memory B-cell repertoires of healthy individuals. In contrast, CD27^+^IgG^+^ B cells from patients who are genetically deficient in IRAK4 or MYD88, which mediate TLR function, include IGHV4-34^+^ clones with decreased frequencies of somatic hypermutation (16). Furthermore, IGHV4-34^+^ IgGs from IRAK4-deficient or MYD88-deficient patients often exhibit unmutated FWR1 motifs, indicating that these antibodies still recognized I/i antigens. On the other hand, antibodies from healthy donors carried FWR1 mutations that abrogated self-reactivity. However, this paradoxical self-reactivity correlated with the binding of these IGHV4-34^+^ IgG clones to commensal bacterial antigens. Thus, B cells expressing germline-encoded self-reactive IGHV4-34 may represent an innate-like B-cell population specialized in containing commensal bacteria when the gut barrier is breached.

B cells with autoreactive BCRs are prevented from secreting autoantibodies through a series of tolerance checkpoints known as “clonal anergy.” Anergic B cells that migrate into the germinal center undergo mutations that reduce self-reactivity, leading to “clonal redemption” and the production of antibodies with exquisite specificity for foreign immunogens (17). Evidently, clonal redemption of anergic B cells as a mechanism of immune tolerance inherently carries the risk of autoimmunity. Reed *et al*. (15) discussed the benefits of this process when they reported that self-reactivity of IGHV4-34 by antibodies was removed by a single somatic mutation that paradoxically decreased binding to the foreign immunogen. They discussed that in the case of T cells, holes in the naive repertoire created by clonal deletion are guaranteed to be different in each individual of the species because of major histocompatibility complex (MHC) polymorphism makes it difficult for microbes to exploit these gaps across the human population. However, in the case of the naive B-cell repertoire, holes created by deletion or editing would be the same in all individuals and in most species. One solution is to keep self-reactive B cells in an anergic state within the naive repertoire and allow them to undergo somatic mutations away from self-reactivity when activated. When these B cells bind to microbial antigens, they can receive help from follicular T helper (Tfh) cells.

## The immunological role of polyreactive antibodies in biological defense

**Figure 1.**
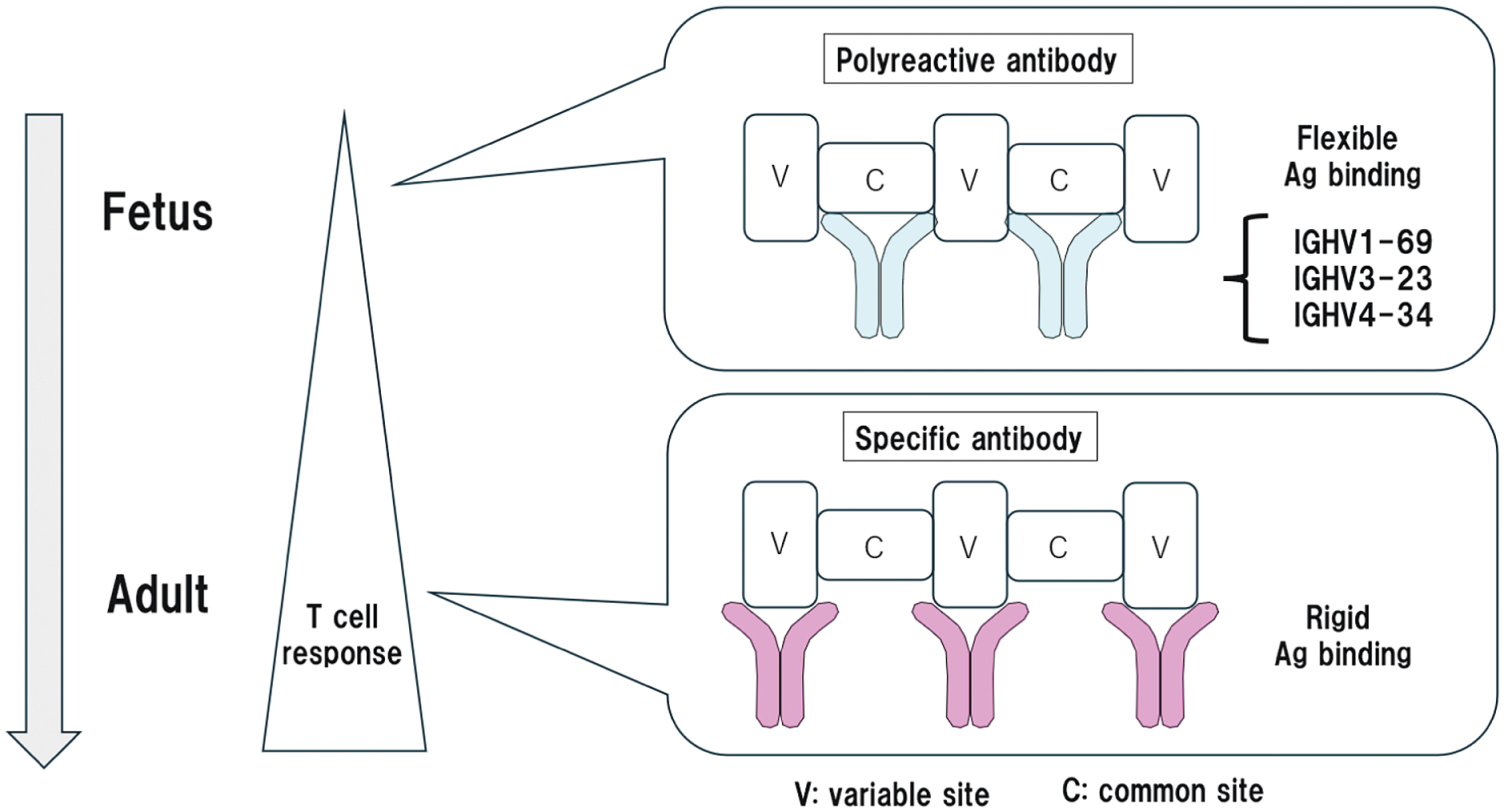
Differences between specific and polyreactive antibodies. During the fetal stage, when T cells are not yet developed, naive B cells expressing polyreactive B-cell receptors are responsible for immune responses. Polyreactive antibodies have a more flexible structure and can bind to common sites. In adults, T cells are developed and memory B cells with specific B-cell receptors differentiate. Specific antibodies have a more rigid structure and bind to variable sites.

The significance of polyreactive antibodies remains unclear. However, accumulating evidence suggests that polyreactive antibodies play an important role in immune responses to microorganisms. Reyes-Ruiz and Dimitrov (18) pointed out that polyreactive antibodies against viruses are highly flexible and capable of recognizing common structures within the *viruses* that are less prone to mutation. The molecular flexibility allows the antibody to overcome constraints by variation of the epitope. Polyreactive antibodies may have the advantage of fewer somatic mutations, enabling a rapid response to mutated viruses. Defensive antibodies against viruses such as influenza, include monoreactive antibodies specific to certain epitopes, and polyreactive antibodies, which bind to common structures (Fig. 1).

Among anti-influenza virus antibodies, those that recognize variable epitopes have high specificity but lose binding ability if the epitope mutates, thereby failing to exhibit protective activity against the virus. In contrast, antibodies that recognize conserved epitopes such as the hemagglutinin (HA) stalk and receptor-binding site (RBS) exhibit polyreactive properties (19). An expansion of antibodies using specific *IGHV* genes, such as *IGHV1-69, IGHV3-23*, and *IGHV4-34*, is observed in human peripheral blood exposed to influenza antigens. The importance of polyreactive antibodies in defense against viruses, such as BK or JC polyomaviruses and human immunodeficiency virus (HIV), has also been reported (20, 21).

Chen *et al*. (22) created over 450 recombinant antibodies cloned from single B cells isolated from the liver, bone marrow (BM), and spleen from four human second-trimester fetuses. Defining antibodies that react with dsDNA, insulin, and lipopolysaccharide (LPS) as polyreactive, they found that polyreactivity was associated with specific *IGHV* genes, namely *IGHV1, IGHV3*, and *IGHV4*. These polyreactive antibodies also showed reactivity to dead cells and commensal bacteria. Because the thymus and T cells are absent in the fetus, naive B cells are responsible for the immune response. These data suggest that during the fetal period, a naive B-cell repertoire useful for infection defense and dead cell clearance is formed, with polyreactive antibodies playing an important role.

## The importance of B cells in the onset and prognosis of SLE

Evaluating B-cell functions in SLE is crucial to understand the pathological roles polyreactive B cells or antibodies play in SLE. In diseases like SLE, integrating clinical data with immunological data enables the identification of immunological parameters associated with prognosis. We integrated robust genomic and transcriptomic information within the immunological data with clinical data. Using cell sorters, we separated peripheral blood from 337 cases with immune-mediated diseases and 79 healthy individuals into 28 types of immune cells and performed RNA sequencing (23). Additionally, we conducted whole-genome sequencing of all samples to construct the functional genome database, ImmuNexUT. The ImmuNexUT is the largest functional genome database for immune cells. Using the expression quantitative trait loci (eQTL) as an annotation for stratified linkage disequilibrium (LD) score regression analysis, allows for the evaluation of the association between genetic risks of various immune-mediated diseases and immune cells. We found that the genetic risk for SLE was most strongly associated with B cells, particularly with B cells in the early stages of differentiation, such as naive B cells and unswitched memory B cells (UnswMB).

Furthermore, we analysed the transcriptomes of 27 types of immune cells from 136 SLE cases and 89 healthy individuals (24). Approximately 25% of the 136 SLE cases were inactive, and about 25% had high disease activity. We attempted to identify the signature defining SLE, by comparing inactive SLE with healthy individuals, which we called the disease state signature. Additionally, by comparing high disease activity SLE with inactive SLE, we aimed to identify the signature associated with high activity, the disease activity signature. Our data showed that the disease state signature was associated with mitochondrial pathways in T cells and B cells and the complement pathway in all immune cells, whereas the disease activity signature was associated with ribosome pathways in all immune cells and cell cycle pathways in T cells and B cells. Interestingly, patients with increased expression of genes related to mitochondrial pathways had increased organ damage. Since organ damage is associated with prognosis, mitochondrial pathways were considered to be potentially related to long-term prognosis.

The importance of B cells in SLE has been elucidated by basic research as mentioned above and has been undeniably confirmed in clinical trials in which anti-CD19 chimeric antigen receptor (CAR)-T cell therapy achieved drug-free remission (25–27).

## The importance of IGHV4-34^+^ UnswMB cells in SLE

To investigate the role of B cells in the pathogenesis of SLE, it is crucial to analyse the BCR repertoire and evaluate polyreactive B cells. We analysed the BCR repertoire of five B-cell subsets derived from 595 cases, including those with immune-mediated diseases and healthy individuals (28). Antigen specificity is speculated to strongly depend on the CDR3 region; however, during the maturation process of B-cell receptors, not only the CDR3 region but also the FWR is subjected to selection pressure (29). In autoimmune diseases, changes in the usage frequency of VH genes have been observed (30), suggesting that regions other than the CDR3 region of the BCR also affect antigen recognition. Therefore, we evaluated the BCR repertoire using the V, D, and J gene usage, which can be accurately assessed. Memory B cells according to their IgD expression could be resolved into CD19^+^CD27^+^IgD^−^ switched memory B cells (SwiMB) and CD19^+^CD27^+^IgD^+^ UnswMB. After antigen recognition in the secondary lymphoid organs, naive B cells differentiate into SwiMB. UnswMB are induced by innate stimuli such as TLR signals. CD27^−^IgD^−^ double-negative B cells (DNB) are mostly mature antigen-experienced B cells originating from the extrafollicular pathway (31). Principal component analysis (PCA) based on V, D, and J gene usage resulted in five distinct clusters. Interestingly, these five clusters corresponded to five B-cell subsets, namely: naive B, UnswMB, SwiMB, DNB, and PB. This suggests that V, D, and J gene usage partially determines B-cell differentiation.

Next, we evaluated *IGHV* gene usage in the five B-cell subsets of healthy individuals and found a dominant set of *IGHV* genes for each subset, because *IGHV* genes demonstrated strong skewing in the PCA because of V, D, and J gene usage. This result was consistent with the findings from the PCA described above. Approximately 30% of human naive B cells are considered self-reactive. In normal human peripheral blood, in which approximately 40% of transitional B cells harbor autoreactivity, the frequency is reduced to 20% when they are differentiated into naive B cells (32, 33). Therefore, the dominant *IGHV* genes in naive B cells may have self-reactive potential. Indeed, the dominant *IGHV* genes in naive B cells included *IGHV1-69* and *IGHV4-34*, which are both autoreactive and polyreactive. Autoreactive B cells are known to have inhibited differentiation into memory B cells, suggesting that autoreactive and polyreactive B cells might also have suppressed differentiation into memory B cells.

When comparing V, D, and J gene usage between memory B cells in patients with SLE and healthy individuals, a group of V, D, and J genes, including *IGHV1-69* and *IGHV4-34*, was used much more frequently in SLE memory B cells. These genes matched those dominant in naive B cells of healthy individuals. Thus, the repertoire dominant in naive B cells in healthy individuals was observed in the memory B cells of patients with SLE. We named this phenomenon “repertoire naiveness”. Additionally, by creating a repertoire naiveness score (RNS) to quantify this phenomenon, we found that the RNS of UnswMB and PB significantly correlated with SLE disease activity (SLEDAI). This suggests that modifications in the B-cell repertoire might contribute to organ damage in SLE.

Belimumab, a molecular-targeted drug used for SLE, is a therapeutic agent against targeting cytokine B-cell activating factor (BAFF), which promotes the differentiation of naive B cells into memory B cells (34). Evaluating the B-cell repertoire before and after belimumab treatment in 22 patients with SLE, we observed a significant suppression of RNS specifically in UnswMB among B-cell subsets, including plasmablasts, and confirmed that this occurred in parallel with improvements in SLEDAI scores and complement levels (28). This indicates that effective molecular-targeted drugs in SLE might target abnormal B-cell repertoires. Furthermore, examining the relationship between SLE disease activity and *IGHV* gene usage, the frequency of *IGHV4-34* in UnswMB strongly correlated with SLEDAI, and belimumab tended to reduce the frequency of *IGHV4-34* in UnswMB. Investigating factors correlating with RNS in UnswMB, we found an indirect association with the type I IFN signature. The type I IFN signature was related to the expression of chemokine (C-X-C motif) ligand 13 (CXCL13) and interleukin (IL)-21 in CD4^+^ T cells, and the expression of these molecules correlated with RNS in UnswMB. A previous report showed that 15% of IgG memory B cells in SLE are autoreactive (35). However, in the analysis of the SLE cohort from ImmuNexUT, the RNS in SwiMB did not decrease when belimumab was effective, and the frequency of *IGHV4-34* on SwiMB did not correlate with SLEDAI.

**Figure 2.**
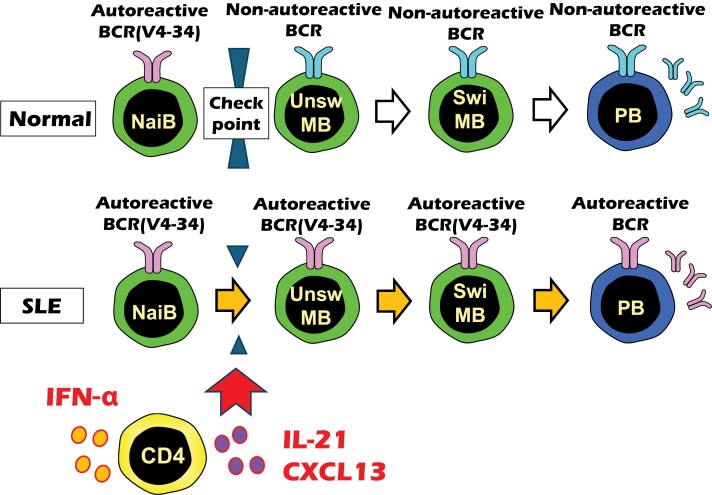
A model of BCR checkpoint regulation induced by type I IFN and CXCL13-producing CD4^+^ T cells. The autoreactive BCR repertoire in SLE was evaluated in 136 patients with SLE (and 89 healthy individuals) using polyreactive *IGHV* genes as an indicator. B cells that normally express self-reactive and polyreactive BCRs stop differentiating at the naive B-cell (NaiB) stage; however, they differentiate into unswitched memory B (UnswMB) cells, switched memory B cells (SwiMB) and plasmablasts (PB) in SLE. Upstream of this differentiation of self-reactive and polyreactive B cells, there is activation of CD4^+^ T cells by type I IFN, resulting in the expression of IL-21 and CXCL13. BCR, B-cell receptor; CXCL13, chemokine (C-X-C motif) ligand 13; IFN, interferon; IL, interleukin; SLE, systemic lupus erythematosus.

From these results, it was hypothesized that in SLE, type I IFN (e.g. IFN-α) induces the production of CXCL13 and IL-21 in CD4^+^ T cells. Subsequently, these factors promote the differentiation of B cells, which would otherwise remain at the naive B-cell stage, into memory B cells (Fig. 2). There are several CD4^+^ T-cell subsets that produce CXCL13 and IL-21, such as Tfh cells (36), peripheral helper T (Tph) cells (37), and age-associated helper T (ThA) cells (38). These T-cell subsets with B-cell helper activity may contribute to the skewing of the B-cell repertoire toward autoreactivity and polyreactivity. Consistently, Arvidsson *et al*. (39) analysed peripheral blood B cells from patients with primary Sjögren’s syndrome by single-cell RNA sequencing, and found differential usage of *IGHV1-69* and *IGHV4-30-4*. Polyreactive B cells may be pathologically relevant in rheumatic diseases associated with increased type I IFN.

## Severe coronavirus disease 2019 (COVID-19) induces antinuclear antibodies through the use of IGHV4-34

Type I IFN is produced in large quantities in SLE and during viral infections (40, 41). If type I IFN induces the differentiation of B cells expressing IGHV4-34, memory B cells and plasma cells expressing IGHV4-34 may also be induced during viral infections. Woodruff *et al*. (42) investigated autoantibodies emerging during the onset of COVID-19 and reported that over 40% of the severely affected cohort admitted to the Intensive Care Unit (ICU) showed reactivity to nuclear antibodies at titers of 1:160 or higher. Recombinant antibodies were created from antibody-producing B cells and selected antibodies reacted to severe acute respiratory syndrome coronavirus 2 (SARS-CoV-2) antigens from two severe ICU cases. Nearly one-third of nucleocapsid or spike protein-reactive antibodies used IGHV4-34, and they included clones with antinuclear antibody activity and other autoreactive characteristics. This indicates that B cells expressing IGHV4-34, which are autoreactive and respond to viral antigens, are activated during viral infections.

## Conclusion

In the human immune system, when effective T cells are absent during the fetal period, polyreactive B cells and antibodies may contribute to the clearance of microbes, dead cells, and self-antigens. This polyreactivity is associated with specific *IGHV* genes such as *IGHV4-34* and *IGHV1-69*. In adulthood, the differentiation of these highly reactive polyreactive B cells is suppressed, and these cells remain as naive B cells. However, in SLE, because of the influence of type I IFN, B cells that would remain as naive B cells in healthy individuals differentiate into memory B cells. Some of these differentiated memory B cells express IGHV4-34, and the proportion of UnswMB expressing IGHV4-34 strongly correlates with SLEDAI. Evaluating polyreactive B cells using IGHV as an indicator in SLE may help to stratify patients and aid in the development of new treatments.
